# The genetics of situs inversus without primary ciliary dyskinesia

**DOI:** 10.1038/s41598-020-60589-z

**Published:** 2020-02-28

**Authors:** Merel C. Postema, Amaia Carrion-Castillo, Simon E. Fisher, Guy Vingerhoets, Clyde Francks

**Affiliations:** 1 Department of Language & Genetics, Max Planck Institute for Psycholinguistics, Nijmegen, The Netherlands; 2 Donders Institute for Brain, Cognition and Behaviour, Radboud University, Nijmegen, The Netherlands; 3 Department of Experimental Psychology, Ghent University, Ghent, Belgium

**Keywords:** Genetic variation, DNA sequencing

## Abstract

*Situs inversus* (SI), a left-right mirror reversal of the visceral organs, can occur with recessive Primary Ciliary Dyskinesia (PCD). However, most people with SI do not have PCD, and the etiology of their condition remains poorly studied. We sequenced the genomes of 15 people with SI, of which six had PCD, as well as 15 controls. Subjects with non-PCD SI in this sample had an elevated rate of left-handedness (five out of nine), which suggested possible developmental mechanisms linking brain and body laterality. The six SI subjects with PCD all had likely recessive mutations in genes already known to cause PCD. Two non-PCD SI cases also had recessive mutations in known PCD genes, suggesting reduced penetrance for PCD in some SI cases. One non-PCD SI case had recessive mutations in *PKD1L1*, and another in *CFAP52* (also known as *WDR16*). Both of these genes have previously been linked to SI without PCD. However, five of the nine non-PCD SI cases, including three of the left-handers in this dataset, had no obvious monogenic basis for their condition. Environmental influences, or possible random effects in early development, must be considered.

## Introduction

Roughly 1:6,000–8,000 people have *situs inversus* (SI), a mirror reversal of the normal asymmetrical arrangement of the viscera^1,2^. SI can occur in combination with Primary Ciliary Dyskinesia (PCD), a recessive genetic disorder which involves mutations that disrupt motile cilia^1^. Cilia are hair-like organelles that protrude from the cell surface into the extracellular space^3^. They are expressed in various tissues including the respiratory epithelium^4^, so that a disruption of ciliary motility can cause symptoms such as chronic bronchitis, inflamed or infected sinuses^5^, which are often present in PCD.

Motile cilia are also expressed early in development, within an embryonic structure called the ‘node’, where they generate a leftward fluid flow that helps to create the left-right body axis^4^. The leftward direction of the nodal flow may be explained by a posterior tilt of the cilia together with their clockwise rotation, arising ultimately from molecular chirality of their component proteins^6–8^. When leftward nodal flow is absent due to recessive PCD-causing mutations, many of which affect component proteins of the ciliary cytoskeleton, it becomes a matter of chance whether the viscera will take up the normal or mirror-reversed positioning^9^. The same principle of randomization appears in mice, for example through recessive mutations in the *Iv* gene^10,11^. Randomization can also explain why some human monozygotic twins are discordant for SI^12^.

However, roughly three quarters of people with SI do not have PCD^1^, and the causes of their SI remain largely unknown. A sibling recurrence risk of 100 times the population prevalence has been classically documented for SI^13^, although it is not clear whether SI individuals with PCD were distinguished from those without PCD when making this calculation, and we have been unable to obtain the primary reference^14^. A few genes have been reported to be involved in SI without PCD, and some of these can also cause partial disruptions of visceral laterality, known as heterotaxy or *situs ambiguus* (SA), including *ZIC3* [MIM:300265]^15,16^*, CCDC11* [MIM:614759]^17^, *CFAP52* [MIM:609804, also known as *WDR16*]^18^, *NME7* [MIM:613465]^19^, and *PKD1L1* [MIM:609721]^20^. The mechanisms by which these non-PCD genes influence visceral laterality are not well understood, but some code for cilia-associated proteins, rather than coding directly for cytoskeletal components of cilia.

A recent exome sequencing study of 323 people with laterality defects included 11 that had SI, while the rest had SA or cardiac malformations such as dextrocardia^16^. Of the 11 people with SI, only one had a clear candidate recessive mutation, in the known SA gene *MMP21*. PCD was not an inclusion criterion for that study, and none of the people with SI had recessive mutations in genes known to cause PCD. One additional participant with SI had a dominant mutation in *ROCK2*, and another had an X-linked mutation in *RAI2* – both of these genes are important for heart development, but it is not clear whether mutations in either were causative for SI^16^. That was the largest previous study, of which we are aware, that screened the exome for mutations in people with non-PCD SI.

Approximately 90% of people are right-handed and have left-hemisphere dominance for language, among other lateralized functions, but the developmental basis for brain asymmetry remains unknown^21–23^. One possibility is that early embryonic mechanisms that give rise to asymmetries of the visceral organs also impact on brain asymmetries. Indeed, such a link has already been established in the *frequent-situs-inversus (fsi)* line of zebrafish^24^. One study of humans reported a possible genetic link between a continuous measure of left-versus-right hand motor skill and genes involved in visceral laterality, based on analyzing genetic variants which are common in the population^25^. However, the sample size of under 3000 subjects was low for complex-trait genome-wide association analysis using common genetic variants. A much larger study of over 300,000 subjects from the UK Biobank found no support for a link of left-handedness to genes involved in visceral asymmetry^26^. The same large study identified an association of a common variant at the microtubule-associated gene *MAP2* with left-handedness, with a small effect^26^.

Intriguingly, the general population rate of 10–15% left-handedness is not altered in SI with PCD^27,28^. This implies a developmental dissociation between brain laterality for hand motor control and nodal-ciliary visceral patterning. However, in the only previous study of handedness in SI to specifically target non-PCD individuals, Vingerhoets *et al*.^29^ recently reported that five of nine non-PCD SI subjects were left-handed. Assuming a population rate for left-handedness of 10%, an exact, one-tailed binomial test gives P = 0.0009 for this elevated rate of left-handedness in non-PCD SI. A further six SI subjects studied by Vingerhoets *et al*.^29^ had PCD – but, as expected, they showed no elevated rate of left-handedness (1 out of 6).

The data of Vingerhoets *et al*.^29^ suggest the existence of developmental mechanisms that link brain asymmetry and visceral laterality, independently of genes involved in PCD, although the small sample size cannot provide proof of this. In fact, a study from 1950 included 270 people with SI that were identified based on mass X-ray photography of the Norwegian population, and left-handedness was not over-represented compared to the general population^12^. The ascertainment method in that study suggests that most SI individuals did not necessarily have PCD, although this was not investigated formally^12^. If most SI individuals in the 1950 study did not have PCD, it would indicate that there is no elevation of left/handedness in non/PCD SI, and therefore that the elevated rate of left-handedness among the nine non-PCD SI participants of Vingerhoets *et al*.^29^ was a chance finding in a small sample, or else an unusual feature of this particular set of subjects. Like the 1950 study, an even older study, from 1938, also reported a normal population rate of left-handedness in SI, although here ‘most of those included were examined on account of illness or a congenital malformation’^30^. In the 1938 study, therefore, it seems possible that many of the SI individuals had PCD, although further information was not given^30^. Left-handedness has a heritability of roughly 25% based on twin and family data^31^, but only around 2% based on genome-wide genotype data for common polymorphisms, within the UK Biobank dataset^32^. However, it has been proposed that left-handedness may sometimes occur due to genetic mutations that are rare in the population, but with substantial effects on brain laterality when present^33,34^. Rare genetic effects are not well captured in genome-wide association or SNP-heritability studies, which are typically based on common variation in the genome^26^.

Here we performed an exploratory whole genome sequencing study in the same set of 15 SI subjects studied by Vingerhoets *et al*.^29^ (six with PCD and nine without), as well as 15 healthy controls matched for age, sex, education, and handedness (Table 1). The primary goal was to identify rare, highly penetrant mutations in the nine people with non-PCD SI, as relatively little is known about the genetic contributions to non-PCD SI. As these nine subjects showed an elevated rate of left-handedness, our study also had the potential to yield novel insights into the developmental origins of left-right patterning of both brain and body. The six SI subjects with PCD were included as positive controls, in which we expected to find recessive mutations in known PCD-causing genes.

**Table 1:** The most likely causal recessive mutations in the fifteen SI subjects.

Subj	SI group	Sex/ Age	EHI	NH	CHD	Daily wet cough	Type	Gene	Clinvar	rs ID	Start position	ref	alt	MAF	AAC	impact
SI02	non-PCD	M/50	0.9	R	0	?	hom	*PKD1L1*	SA | SI	rs528302390	47870810	T	TCA	0.00109	—	splice donor
SI03	non-PCD	F/26	−0.8	L	0	yes		unsolved								
SI04	non-PCD	M/23	−1	L	1	no		unsolved								
SI05	non-PCD	M/27	0.9	R	0	no		unsolved								
SI07	non-PCD	F/35	0.9	R	1	?		unsolved								
SI09	non-PCD	F/36	0.7	L^§^	0	no		unsolved								
SI12	non-PCD	F/40	0.9	R	0	no	chet	*DNAH5*	Kartagener	None	13900415	A	C	—	E/*	stop gained
							chet	*DNAH5*	Kartagener	None	13769691	G	GC	0.00002	A/X	frameshift
SI14	non-PCD	M/18	−0.8	L	1	no	chet	*CFAP52/WDR16*	SI	rs148905544	9489207	T	A	0.00066	H/Q	Missense variant
							chet	*CFAP52/WDR16*	SI	rs374035006	9536224	C	T	0.00012	R/*	Stop gained
SI16	non-PCD	M/21	−1	L	0	no	hom	*CCDC151*	Kartagener	rs765121016	11533429	T	TCTC	0.00011	E/−	inframe deletion
SI06	PCD	M/46	1	R	0	yes	hom	*LRRC6*	Kartagener	rs767624733	133687728	T	C	0.00017	—	splice donor
SI08	PCD	F/23	0.9	R	0	yes	hom	*DNAH11*	PCD	rs373706559	21659620	A	C	0.00006	Y/*	stop gained
SI11	PCD	F/32	0.9	R	0	yes	chet	*DNAAF1*	Kartagener	rs569633512	84203963	C	T	—	—	splice donor
							chet	*DNAAF1*	Kartagener	rs373103805	84193302	G	C	0.00023	N/K	missense
SI13	PCD	M/48	0.6	L^§^	0	yes	hom	*CCDC114*	Kartagener	rs779459076	48814907	CACG	C	0.00093	−/R	Inframe insert
SI15	PCD	F/31	0.7	R	0	yes	chet	*DNAH5*	Kartagener	None	13786289	A	T	—	K/*	stop gained
							chet	*DNAH5*	Kartagener	rs397515540	13753397	G	GA	0.00034	D/X	frameshift
SI17	PCD	M/39	0.5	R	0	yes	chet	*DNAH5*	Kartagener	rs548521732	13839638	C	T	0.00021	—	splice acceptor
							chet	*DNAH5*	Kartagener	rs769458738	13753597	T	C	0.00004	R/H	missense

Subjects shown with two mutations have possible compound heterozygous mutations, otherwise they have homozygous mutations. The Genome Reference Consorium (GRC) build 37 decoy version was used as reference sequence. EHI: Edinburgh Handedness Inventory score; NH: natural handedness; CHD: Congenital Heart Disease. Type: type of genetic mutation, i.e., homozygous (hom) or compound heterozygous (chet); MAF: minor allele frequency in population databases, if known; AAC: amino acid change. ^§^Self-identified natural lefthander made to convert to right-handedness.

## Methods

### Participants

Table 1 gives an overview of the participants and their characteristics. Fifteen people with radiologically documented SI (mean age = 33 years, 8 males), and 15 controls with normal *situs* matched for age, sex, education and handedness (mean age = 33 years, 8 males), were recruited from Ghent University Hospital and Middelheim Hospital Antwerp. All participants were tested and scanned at Ghent University Hospital following ethical approval by the “Commissie voor Medische Ethiek, Universitair Ziekenhuis Gent”, study approval number 2012/073. All participants gave informed consent for DNA sample collection and genomic analysis in relation to body, brain and behavioural laterality. All methods were performed in accordance with the relevant guidelines and regulations.

Details about the recruitment, diagnosis and selection/exclusion procedure have been described previously^29^. Radiological information (RX or CT) of thorax and complete abdomen was available for eight SI participants, and of thorax and upper abdomen in seven SI participants. The medical reports confirmed that all 15 participants presented with radiologically documented SI.

In five participants with SI, a formal diagnosis of PCD or Kartagener syndrome was found in their medical records. Kartagener syndrome refers to the clinical triad of SI, chronic sinusitis, and bronchiectasis^9^. A sixth SI-participant was identified on account of a radiological consultation regarding infertility. The participant also presented with chronic sinusitis and mild chronic bronchitis. Although no formal diagnosis of PCD was obtained in this case, the presence of chronic upper and lower respiratory infection and infertility in an individual with SI warrants suspicion of Kartagener syndrome. Consequently, we included the participant within the PCD group. In addition, all six members of the PCD group reported having a daily wet cough, and had PICADAR scores of between 8 and 12^35^, and thus predictive probabilities of having PCD of between 66% and 99%, based on this recently-developed, questionnaire-based tool (note that five of these subjects anyway had formal medical diagnoses).

A seventh SI subject (SI03) had no medical record pertaining to PCD but did report daily wet cough. This subject had a PICADAR score of 8. In the study of Vingerhoets *et al*. this SI subject was classified as non-PCD, before the PICADAR assessment was available^29^. For the purposes of the present study, we also treated this subject as non-PCD given the lack of formal medical history of PCD and the ambiguous PICADAR score, as well as the fact that we found no likely recessive mutations in PCD-causing genes in this subject (see Results).

Eight other SI subjects had no medical record of PCD or PCD-like symptoms, and were classified as non-PCD SI cases. Six of these reported no daily wet cough, and two did not answer in this regard. PICADAR scores can only be calculated in the presence of daily wet cough, so that none of these eight cases received PICADAR scores. Three of these cases had been previously diagnosed with congenital heart disease that required surgical treatment, and their radiological files all referred to their cardiac condition. Congenital heart disease is a frequent comorbidity of SI, as the cardiac circulation appears particularly sensitive to perturbation in normal left-right positional information^36^.

Handedness was assessed using the Edinburgh Handedness Inventory (EHI)^37^. All participants had EHI scores >|0.5| (Table 1), so that none were ambidextrous^38^. Note that one non-PCD SI subject reported being forced to switch from left- to right-handedness in childhood, in which case five out of nine non-PCD SI cases were naturally left-handed. One of the six cases with PCD also reported forced left-to-right switching, otherwise the rest were right-handed (Table 1).

### Whole genome sequencing (WGS) and Pre-processing

DNA was extracted from saliva samples using the Oragene kit (Oragene). WGS was performed by Novogene (Hong Kong) using Illumina high throughput sequencing (HiSeq-PE150), creating paired end reads with a length of 150 base pairs (bp). Raw reads, stored in BAM files, were aligned to the human reference genome (the extended “decoy” version of b37) using Burrows-Wheeler Aligner (BWA) software^39^, and sorted and reordered using SAMtools (v1.3.1)^40^. PCR duplicates, which could arise during cluster amplification, were marked using Picard (v2.9.0). Genome Analysis Toolkit (GATK v3.7)^41^ software was used to realign reads around insertions/deletions (indels) and to recalibrate base quality scores per sample.

### Variant calling and quality control

Indels (insertion/deletions) and single nucleotide polymorphisms (SNPs) were called as recommended by the GATK Best Practices workflow. Variant Quality Score Recalibration (VQSR) was performed, and variants with a high probability of being false positive were flagged according to their sensitivity tranche (90, 99, 99.9 and 100). All SNPs and indels within a VQSR tranche of 99.9% or higher were discarded. Variants with a quality depth ≤9 or a call rate ≤0.8 were also removed. Vcftools v1.13 was used to create a summary table in the Variant Call Format (vcf). A total of 13,989,941 SNPs and indels were identified across the 30 subjects, with an average number of 5,186,055 (min = 5,053,188, max = 5,272,561) alternative alleles per subject (i.e. different to the reference genome, build37 decoy version).

### Genomic-level evaluation

For overall descriptive analysis of the participant genomes, a subset of 40,387 variants distributed genome-wide was used that had known Minor Allele Frequencies (MAFs) >0.1, and were the result of pruning to be in low linkage disequilibrium with one another. For this, the flag–indep-pairwise function in Plink (v.1.90b3w)^42^ was applied with a pairwise linkage disequilibrium r^2^ greater than 0.2, based on a SNP-SNP correlation matrix of 1500 by 150 in window size. The resulting data were then used as the basis for inferring pairwise identity by descent (IBD) sharing between subjects (i.e. genetic relatedness), inbreeding, and inconsistencies with reported sex, using the Plink operations–genome,–ibc/het, and–check-sex.

A different subset of 61,467 independent variants distributed genome-wide was used for visualizing population structure through Multi-Dimensional Scaling (MDS) analysis with respect to the 1000 genomes reference dataset (v37)^43^ (downloaded from: ftp://climb.genomics.cn/pub/10.5524/100001_101000/100116/1kg_phase1_all.tar.gz) using the Plink operation –mds-plot.

### Annotation and filtering of single nucleotide variants affecting protein sequences

Variants were normalized using the software tool *vt normalize* (v0.5772–60f436c3)^44^, which ensures consistent representation of variants across the genome. Normalized SNVs were annotated using Annovar^45^ and Variant Effect Predictor (v88)^46^. Gemini (v 0.20.0) was used to select protein coding variants with ‘MEDIUM’ or ‘HIGH’ impact severity annotations, as well as non-coding variants with ‘HIGH’ impact severity annotations (in practice those altering splice donor or acceptor sites). Additional filtering was done in R and comprised the removal of synonymous variants, and of ‘MEDIUM’ variants with a PolyPhen^47^ prediction of ‘benign’ unless annotated as ‘deleterious’ by Sift^48^, and those annotated as ‘tolerated’ by Sift unless predicted to be ‘probably damaging’ by PolyPhen. Data were then processed and analyzed separately under recessive and dominant models:

#### Recessive model

For the recessive model we further excluded variants with a known population frequency ≥ 0.005 in any of the following databases: GNOMAD^49^, ESP^50^, 1000 Genomes^43^ and ExAC databases^49^. The remaining low-frequency variants were considered as putative mutations. Gene-level mutation counts per subject were then made, with a given gene being assigned as recessively mutated when it carried two copies of the same mutation (homozygous) or two different mutations (possible compound heterozygous). Integrative Genome Viewer (IGV v2.3.97)^51^ was used to visualize the possible compound heterozygous mutations, and genes carrying these were discarded as recessive candidates when both mutations were definitely present on the same allele (i.e. “in phase”) on a given sequence read. Finally, genes recessively mutated according to these criteria in any of the fifteen unaffected control subjects were excluded as being potentially causative in cases, and also removed for the purposes of Gene Set Enrichment Analysis (GSEA; see below): this step had the advantage of removing false variants from potential technical artifacts, or variants which are common in the population but not annotated as such in on-line databases. In total, 17 genes were excluded based on overlap with the unaffected control subjects. Genes on chromosome X were processed as part of the recessive pipeline, such that females would need to carry two mutations in a given gene, and males one mutation.

#### Dominant model

A maximum population frequency threshold of 5 × 10^−5^ was applied in this case, and genes carrying at least one rare variant according to this criterion were considered as potentially causative. Again, genes mutated according to this criterion in any of the fifteen unaffected control subjects (N = 47 genes) were excluded as being potentially dominantly causative in cases, and removed for the purposes of GSEA analysis (below).

### Gene set enrichment analysis

To test whether a list of mutated genes in a given set of subjects contained functionally related genes across subjects, we performed Gene Set Enrichment Analysis (GSEA) using the g:Profiler R package (version 0.6.1)^52^. A gene classification scheme derived from the Gene Ontology (GO) database^53,54^ was used. This specified a total of 6380 functionally defined gene-sets, based on a background of 19,327 genes with functional annotations. Many genes are not represented in the GO, particularly mRNA transcripts of no known function or homology, so that the numbers of mutated genes in a given set of subjects was always higher than the subset used as input for GSEA.

Mutated genes on chromosome X were combined with recessively mutated autosomal genes for the purposes of GSEA. GSEAs were performed separately for the mutated gene lists in SI subjects with PCD (54 genes, of which 40 are in the GO), non-PCD SI subjects (60 genes, 38 in the GO), left handed non-PCD SI subjects (42 genes, 26 in the GO), and non-PCD SI cases that were unsolved under the recessive model (27 genes, 13 in the GO).

For the dominant model, GSEA was performed separately for mutated genes in the non-PCD SI subjects (330 genes, 271 in the GO), the subset of non-PCD SI subjects that remained unsolved under the recessive model (183 genes, 148 in the GO), and the left handed subset of non-PCD subjects (201 genes, 163 in the GO). PCD subjects were not tested under a dominant model, as PCD is known to be a recessive phenotype.

In order to confirm that unaffected controls showed no significant pathway enrichment among their mutated genes, a mirrored exclusion was performed whereby any genes mutated in cases were excluded from the control gene lists. This resulted in 56 genes exclusively mutated in controls for the recessive model (of which 34 genes are in the GO), and 533 genes exclusively mutated in controls under the dominant model (440 in the GO).

The identities of genes were first converted using the g:Convert tool^52^ to ensure recognition by the GO schema. The following settings were then used for GSEA: minimum set size = 15, maximum set size = 500, minimum intersect number = 2, hierarchical filtering = moderate. P-values were corrected for multiple testing across gene sets, based on the gSCS correction method in g:Profiler, separately for each input list of mutated genes corresponding to a given set of subjects. This method of multiple testing correction takes into account the hierarchical structure of the sets^52^. We applied a cut-off P-value of adjusted 0.01.

### Candidate gene lists

We queried the genetic data in R (v3.3), based on searching the Clinvar^55^ column of our annotated variant data for the terms: “*situs inversus*”, “*heterotaxy* | *heterotaxia* | *situs ambiguus*”, “PCD | ciliary dyskinesia | Kartagener”, “left-right”, and “asymmetry | laterality”.

A candidate gene list was also created which included genes from the literature that were suggested to be associated with human laterality phenotypes and/or ciliopathies, but not (yet) present in our initial list of candidate genes (Supplementary Table S1). Specifically, a list of ciliopathy genes from a review of this topic^5^ was searched for the same terms as mentioned above plus ‘congenital heart disease’, yielding 57 candidate genes. Additionally, a list of genes potentially associated with human laterality disorders, as compiled in a 2015 review^1^, resulted in the addition of 38 candidate genes. Five more genes were included that were considered as possibly causal of laterality defects in a recent exome sequencing study^16^ (see Introduction). Eleven genes containing variants that were previously reported to associate with measures of hand preference^26^ or *planum temporale* asymmetry^56^ were also added to the list of candidate genes. Finally, 47 more genes – arising from a search for the ‘*situs inversus*’ phenotype - were retrieved from the Mouse Genome Database (MGD)^57^ at the Mouse Genome Informatics website, the Jackson laboratory, Bar Harbor, Maine. (URL: http://www.informatics.jax.org) [Oct, 2017].

### Structural variants

For all participant genomes, structural Variants (SVs) (>50 kilobases in length) were called using a combination of two SV calling algorithms: CNVnator (v0.3.3)^58^ and Lumpy (v 0.2.13)^59^. These algorithms complement each other regarding the kinds of signals in WGS data that they can detect, as CNVnator is based on read-depth, whereas Lumpy is based on paired-end mapping^60^. For CNVnator the bin size was set to 100 base pairs for all genomes except for two, where it was 150 base pairs (we determined a roughly optimal bin size for each subject’s genome, such that that the average read depth for that genome would be at least 4 standard deviations from zero). Lumpy was run using default parameters in “lumpyexpress”.

SVs were then annotated using SV2^61^. Variants that were present in any of the 15 healthy controls were removed from consideration as potentially causative for SI. Only variants that passed the default SV2 filtering criteria for quality were included^61^.

## Results

### Protein-altering single nucleotide variants

#### Recessive mutations

Our variant calling, filtering and annotation pipeline produced between 5 and 15 recessively mutated genes per SI subject. We included six SI subjects with PCD as positive controls, in order to ensure that the variant calling and mutation definition criteria were well calibrated. As PCD is known to be a recessive phenotype for which at least 30 different genes have already been identified^1^, we expected most, or all, of these six subjects to have identifiable mutations in known PCD-causing genes. As expected, each of these six cases had just one recessively mutated gene which was annotated ‘Kartagener’ or ‘PCD’ in the Clinvar database^55^, and was therefore the most likely monogenic cause for their condition (Table 1). These genes were *LRRC6* [MIM:614930], *DNAH11* [MIM:603339], *DNAAF1* [MIM:613190], *CCDC114* [MIM:615038], and *DNAH5* [MIM: 603335] (the latter mutated in two SI cases with PCD, consistent with *DNAH5* being the most common cause of PCD in European-ancestry populations^62^) (Table 1). The PCD subject with a homozygous *LRRC6* mutation (subject SI06) was the only individual to show an elevated inbreeding coefficient and non-European ancestry (Supplementary Table S2, Supplementary Fig. S1).

None of the fifteen unaffected control subjects had any recessively mutated genes annotated ‘Kartagener’, ‘PCD’, ‘SA’ or ‘SI’ in Clinvar.

Surprisingly, two of the nine non-PCD SI cases had recessive mutations in genes annotated ‘Kartagener’ in the Clinvar database^55^. These were subject SI12 (again involving mutations in *DNAH5*), and subject SI16 (in *CCDC151* [MIM:615956]) (Table 1). As these subjects had no medical records pertaining to PCD-like symptoms, and no daily wet cough, then the findings suggest reduced penetrance for PCD.

Two of the nine non-PCD SI subjects had recessive mutations in genes annotated in Clinvar with ‘*heterotaxy*’ and/or ‘*situs inversus totalis’*, but not annotated as PCD-causing (Table 1). These genes were *PKD1L1* [MIM: 617205] in subject SI02, and *CFAP52* [MIM: 609804] in subject SI14. A homozygous missense mutation in *PKD1L1* was previously reported in an individual with SI and congenital heart disease but no PCD, as well as recessive splicing mutations in two individuals with heterotaxy^20^. Our subject SI02 had no diagnosis of congenital heart disease (CHD), and was right-handed (Table 1). As *PKD1L1* is a known recessive cause of non-PCD SI, we consider this gene to be the most likely cause of the non-PCD SI in subject SI02. As regards *CFAP52* (also known as *WDR16*), a homozygous deleterious deletion was previously reported to segregate with *situs* anomalies, including heterotaxy and *situs inversus totalis*, in a consanguineous family^18^. SI14 carried possible compound heterozygous mutations in this gene (Table 1), which are therefore the most likely causes of non-PCD SI in this individual. SI14 is left-handed and has CHD (Table 1).

Subject SI03, who had no formal medical history of PCD, but had reported an intermediate PICADAR score, showed no recessive mutations in known PCD genes, which tends to support non-PCD status for this subject. This meant that five non-PCD SI cases did not have recessive mutations in genes known to cause human laterality disorders, as annotated in Clinvar, and therefore remained ‘unsolved’ under a recessive model (Table 1). Among these five non-PCD SI cases, three were left-handed, and were therefore of primary interest for the present study. Three of these same subjects also had CHD (Table 1).

We constructed a list of known or suspected laterality genes with reference to the literature and mouse laterality phenotypes (Methods; Supplementary Table S1), but none of these genes had recessive mutations in the five unsolved non-PCD SI subjects.

We note possible compound heterozygous mutations in *PKD1* [MIM:601313], as a paralogue of *PKD1L1* [MIM:609721], in subject SI14 (Supplementary Table S3). However, mutations in this gene would be expected to cause autosomal dominant Polycystic Kidney Disease^63,64^, and since there was no such diagnostic record for this subject, one or both of these specific mutations probably has limited functional impact and is therefore unlikely to be a monogenic cause for SI either. As noted above, SI14 anyway had likely causative mutations in *CFAP52*.

*KIF13B* [MIM:607350] was putatively recessively mutated in subjects SI12 (likely solved through *DNAH5*) and SI14 (likely solved through *CFAP52*) (Supplementary Table S3). Although no literature has linked *KIF13B* to PCD or laterality phenotypes, *KIF3A* [MIM:604683], another kinesin encoding gene, is known to be involved in LR determination^65^. Moreover, *KIF3B* [MIM: 603754] is known to affect motility of nodal cilia and LR determination^66^. Together with dyneins, kinesins allow ciliary proteins to enter the organelle via the transition zone by transporting them as cargo^67,68^, and thereby play an important role in ciliary construction and maintenance^67,68^. The mutations in *KIF13B* might therefore conceivably have affected phenotypic outcomes in subjects SI12 and SI14, but we cannot confidently assign roles to *KIF13B* on the basis of this evidence.

#### Dominant mutations

For the five non-PCD SI cases who remained unsolved under a recessive model, we also considered a dominant model using a maximum known mutation frequency of 5 × 10^−5^, and again cross-referenced the mutated genes against Clinvar and our candidate gene list (Supplementary Table S1), but no likely causative genes emerged (Supplementary Table S3) (see Discussion).

Subject SI05 showed a heterozygous mutation in *LRRC6* [MIM:614930] (Supplementary Table S3), which was included among our candidate genes. However, since recessive – but not dominant - mutations in this gene have been associated with PCD^69^, it is unlikely that this mutation is causal for non-PCD SI in this subject.

We also note a heterozygous mutation in *WDR62* [MIM:613583] in the unsolved case SI09 (Supplementary Table S3). Although mice with mutations in this gene have shown dextrocardia and right pulmonary isomerism (MGI:5437081)^57^, humans with recessive *WDR62* mutations do not show altered laterality. Instead, they have shown infantile spasm, microcephaly and intellectual disability^70^. It is therefore unlikely that *WDR62* is a dominant cause of altered laterality in humans.

The same subject SI09 also had a heterozygous mutation in *PLXND1* [MIM:604282] (Supplementary Table S3), a gene which appears among search results for the phenotype ‘*situs inversus*’ within the Mouse Genome Database^57^. However, while *PLXND1* is annotated as a cause of aortic arch and atrial abnormalities in this database, it is not annotated with *situs inversus* or heterotaxia, so that the basis for the search result is unclear. We did not find evidence for this gene’s involvement in visceral laterality in a further literature search.

Subject SI03 had a heterozygous mutation in *SPEF2* [MIM:610172] (Supplementary Table S3). Loss of *SPEF2* function in mice results in spermatogenesis defects and primary ciliary dyskinesia^71^, but this is through recessive mutations, so that we do not consider the heterozygous mutation in SI03 as a strong causal candidate for non-PCD SI.

### Gene set enrichment analysis

We first performed gene set enrichment analysis using the positive control set of six SI subjects with PCD. As noted above, the six PCD subjects had likely recessive monogenic causes in five different genes (two subjects had mutations in the same gene, *DNAH5*). As expected, from the total combined list of recessively or X-linked mutated genes in these six subjects, of which 40 genes were included in the GO schema, we obtained significant results for various cilia-related pathways, such as ‘axoneme’ (p = 0.004), ‘outer dynein arm assembly’ (p = 3.85 × 10^−5^), ‘dynein complex’ (p = 4.89 × 10^−5^), and ‘microtubule based movement’ (p = 1.41 × 10^−4^) (Table 2) (all P-values adjusted for multiple testing across gene sets, see Methods). When the single most likely causative gene for each PCD subject was removed from the gene list, which left 36 recessively or X-linked mutated genes in these subjects that are present in the GO schema, there were no longer any significant enrichment terms, which further supports that the monogenic causes had been correctly identified in these subjects. The gene set enrichment analysis in the PCD subjects confirmed that, despite a relatively small number of subjects (i.e. six), the analysis was well powered to identify affected biological processes, even when most individual subjects had monogenic causes in different genes, and each subject had other, non-causative mutated genes.

**Table 2:** Gene set enrichment analysis under a recessive mutation model.

Group	p-value^a^	term size	query size^b^	overlap size	term ID	term name	intersection	samples	Pval Fisher^c^
Non-PCD SI (n=9)^d^	NS		38						
Non-PCD SI LH (n=5) ^d^	NS		26						
Unsolved cases (n=5^d^	NS		22						
SI with PCD (n=6)	3.85 × 10^−5^	17	40	4	GO:0036158	outer dynein arm assembly	DNAH5, CCDC114, LRRC6, DNAAF1	SI06, SI11, SI12, SI13, SI15, SI17	7.71 × 10^−13^
SI with PCD (n=6)	1.41 × 10^−4^	296	40	8	GO:0007018	microtubule-based movement	DNAH5, CCDC114, DNAH11, WDR60, LRRC6, DNAAF1, FMN2, DNAH12	SI06, SI08, SI11, SI12, SI13, SI15, SI17	9.74 × 10^−15^
SI with PCD (n=6)	4.89 × 10^−5^	48	40	5	GO:0030286	dynein complex	DNAH5, CCDC114, DNAH11, WDR60, DNAH12	SI08, SI11, SI12, SI13, SI15, SI17	1.68 × 10^−13^
SI with PCD (n=6)	0.004	113	40	5	GO:0005930	axoneme	DNAH5, CCDC114, DNAH11, DNAAF1, RP1L1	SI03, SI08, SI11, SI12, SI13, SI15, SI17	1.38 × 10^−11^
SI with PCD (n=6)	0.002	427	40	8	GO:0099568	cytoplasmic region	DNAH5, CCDC114, DNAH11, SHROOM2, DNAAF1, FMN2, RP1L1, PCLO	SI03, SI06, SI08, SI11, SI12, SI13, SI15, SI17	1.88 × 10^−13^
Unaffected controls (n=15)	NS		34						

LH: left-handed. ^a^P-values are corrected for multiple testing using the gSCS method. NS: no significant gene sets. ^b^The number of mutated genes present in the GO schema. ^c^Uncorrected P-values were calculated using Fisher’s exact test. ^d^Results were the same after excluding subject SI03.

With this in mind, we then performed gene set enrichment analysis in the non-PCD SI subjects, who were of primary interest for the present study. However, no significant enrichments were found when testing the list of recessively or X-linked mutated genes in the nine subjects with non-PCD SI, of which 38 genes were included in the GO schema. There was also no significant functional enrichment when testing the recessively mutated or X-linked genes in the five left-handed subjects with non-PCD SI, of which 26 genes were in the GO schema, and nor when testing the list of genes in the five unsolved non-PCD SI subjects, of which 12 genes were present in the GO schema (Table 2). In addition, gene-set enrichment analysis with dominantly mutated genes as input did not produce significant results in the non-PCD SI cases, nor the left-handed or unsolved subsets.

As expected, the lists of recessively/X-linked and dominantly mutated genes in the fifteen unaffected control subjects did not produce any significant gene set enrichment terms (Table 2).

### Structural variant analysis

We additionally screened the genomes of all subjects for structural variants (such as larger-scale deletions and duplications), using two complementary algorithms (see Methods). SI subjects had SVs affecting an average of 9.7 genes per subject (min = 2 SVs, max = 16 SVs), and controls had SVs affecting an average of 9.6 genes per subject (min = 5 SVs, max = 16 SVs). None of the SI subjects, regardless of PCD status, had SVs affecting genes that were annotated SI, PCD, Kartagener, *situs ambiguus* (SA), or Heterotaxy (HTX) in Clinvar, nor affecting genes from our list of candidate laterality genes.

## Discussion

In this study we aimed to identify rare, highly penetrant genetic mutations that cause SI without PCD, by analysing whole genome sequence data from nine SI subjects without medical histories of PCD, five of whom were left-handed. We additionally included six SI subjects with PCD as positive controls, and fifteen unaffected subjects as negative technical controls.

Likely monogenic causes were identified in all positive controls, i.e. each of the six PCD subjects had one recessively mutated gene (usually a different gene in each subject) that is already known to cause PCD when mutated. The six PCD subjects also confirmed that significant pathway enrichment could be detected on the basis of their mutated gene lists, as multiple gene sets related to ciliary functions were detected. The fifteen unaffected control subjects showed no recessive mutations in genes known to cause PCD or laterality phenotypes, as expected.

Two of the nine non-PCD SI subjects had likely recessive monogenic causes in known PCD genes. This may indicate reduced penetrance of these mutations for PCD-like symptoms, such as lung symptoms, although they can apparently affect *situs* in early development. One non-PCD SI subject, who was right-handed, had a homozygous mutation in a gene already known to cause SI without PCD when mutated, i.e. *PKD1L1*^20^. This gene encodes a component of a calcium channel which is associated with non-motile cilia^72^. Another non-PCD SI subject, who was left-handed and had CHD, had compound heterozygous mutations in *CFAP52*, which is also known as a cause of either heterotaxy or SI without PCD, when mutated^18^. CFAP52 protein has been proposed to play a role in cilia-related signal transduction processes^73^.

However, the five remaining non-PCD SI subjects had no obvious monogenic basis for their condition, i.e., they did not have likely causative mutations in genes known to cause human laterality disorders as annotated in the Clinvar database, or within a list of known or suspected laterality genes based on literature searching and mouse phenotypes. Our findings are similar to the findings from Li *et al*.^16^, whose exome sequencing study of laterality defects included 11 subjects with SI, of which 8 had no likely candidate mutations (see Introduction).

Furthermore, gene set enrichment analysis of the mutated genes in non-PCD SI subjects did not identify significant biological pathways, in either the whole set, or the left-handed subset, or the subset that was ‘unsolved’ by recessive monogenic causes. In other words, the biology of their non-PCD SI could not be linked via the mutations that they carried. Finally, we also considered larger genomic rearrangements known as Copy Number Variants (CNVs), but no obvious candidate genes emerged. A monogenic model is still possible for the majority of the non-PCD SI cases, but would have to involve genetic heterogeneity across a set of genes which are not currently linked in terms of their known biology in the Gene Ontology, at least to an extent that was discernible in this dataset, as it was for the PCD subjects.

Among the five ‘unsolved’ non-PCD SI subjects, three were left-handed, and therefore comprised the majority of left-handed non-PCD SI subjects in this study. This meant that we did not identify a genetic-developmental pathway that links handedness and visceral asymmetry in this study. The question of whether, and how, handedness is linked developmentally to visceral laterality in humans remains unanswered. As noted in the Introduction, the existing literature remains ambiguous, and on balance does not support overt links^12,22,25–30^. It is possible that continuous measures of lateralized hand performance, such as pegboard measures or hand grip strength, might reveal stronger links with genes involved in visceral laterality^25^. However, we had no such measures available in the present study.

Some studies based on small samples have suggested that aspects of brain structural asymmetry can be altered in SI^74–77^. Indeed, this was previously assessed in the same SI subjects and controls as analyzed in the present study^29,78^ - briefly, *planum temporale* and arcuate fasciculus asymmetries were not altered in SI, but the overall cerebral torque showed evidence of left-right reversal in non-PCD SI. At that time there was no genetic information available for these individuals. In light of the genetic data in the present study, the group of nine non-PCD SI individuals has now fragmented into two with likely low-penetrance PCD, two with likely causative mutations in non-PCD-causing genes, and five genetically unsolved. We do not attempt here to do group-based statistics with quantitative measures of brain structural asymmetry in these very small groups. However, this should be a line of research as larger datasets become available.

In the present study we focused on rare, protein-coding mutations, but genetic contributions to non-PCD SI might also involve non-coding variation, or rare combinations of multiple common variants. The noncoding genome comprises 98% of the genome, but interpreting the variation within these regions is challenging. Several attempts have been made to rank potentially causative variants across the genome based on scores that integrate different types of information, including conservation of DNA sequence, regulatory information^79^, and population genomic data^80–85^. However, these ranking approaches are currently not very concordant with each other^86^. For the present study we did not address these possibilities, which must await larger sample sizes and an improved understanding of the role of rare, non-coding mutations in phenotypic variation.

*In utero* environmental effects such as prenatal drug exposure might also affect left-right determination^87^. As regards handedness, this is known to associate with various early life factors including birthweight and breastfeeding, although not to a degree which is remotely predictive at the individual level^88^. As noted in the introduction, left-handedness has a heritability of roughly 25% in family and twin studies, and lower in SNP-based heritability studies. Regardless, the primary causes of this trait remain unknown.

In this study there was a degree of diagnostic uncertainty as regards the PCD status of some SI subjects. However, it was not the purpose of the present study to achieve a clinical diagnosis of the presence or absence of PCD, nor to confirm already-known PCD genes. In this context we did not, for example, sequence the mutations in the PCD subjects by another technique for validation, nor confirm allelic phase in the compound heterozygotes. Rather, we were concerned to identify potentially causative mutations in the nine SI subjects without medical histories of PCD, who also showed an elevated rate of left-handedness, with the potential to yield important basic insights into body and brain laterality. If we had found clear candidate mutations in genes not previously linked to PCD or SI, then further validation and functional characterization of those mutations would have been appropriate, but this did not arise.

Regardless of the PCD status of any individual SI subject in this study, the overall pattern of results was clearly different between the PCD and non-PCD groups, where all six positive control subjects in the PCD group had mutations in known PCD-causing genes, while five out of nine in the non-PCD group had no obvious monogenic mutations. Also, the pathway analyses in various different subsets of the non-PCD SI group showed a consistent lack of significant enrichment, whereas clear signals emerged from the PCD group, which further supports an overall distinction of the groups. Nonetheless, further detailed investigation of subjects SI12 and SI16 with PCD diagnostic tools might reveal lung and other ciliary symptoms to an extent, as these two subjects had likely causative mutations for their SI in known PCD-causing genes^89^.

Although ciliary-induced nodal flow plays a crucial role in symmetry breaking, some organisms, such as chicks and fruit flies (*Drosophilia melanogaster*), do not have motile nodal cilia, yet they do show asymmetrical organs^90^. For example, left-right asymmetry in chicks involves rearranging the relative orientations of cells expressing critical genes at the node, in a non-ciliary-dependent manner^91^. Furthermore, left-right asymmetry of the *Drosophilia* gut is established by intracellular cytoskeletal organization that may give rise to cellular shape chirality, by means of unidirectional tilting of radial fibers, and anti-clockwise swirling of transverse fibers^92^. Similarly, the actin-related *Lsdia1* gene affects shell coiling direction in the water snail^93^. Whether such mechanisms also influence left-right organ asymmetry in mammals is unclear. In the present study we saw no mutations in the orthologues of two genes implicated in cellular chirality in *Drosophilia*, *MYO1D* or *MYO1C*^94^, nor in the orthologues of two genes thought to affect laterality in chicks and frogs, *SLC6A4* and *SLC18A2*^95^. We were not able to identify a human homologue of *Lsdia1*.

Future studies in larger human cohorts may help to identify more genetic contributions to non-PCD SI, and clarify the extent to which developmental mechanisms might be shared with brain asymmetry.

## Supplementary information

Supplementary Information.

## Data Availability

Requests to access the genomic datasets generated for the current study will be considered in relation to the consents, relevant rules and regulations, and can be made via the corresponding author.
